# IgG4-Related Inflammatory Pseudotumor Involving the Clivus: A Case Report and Literature Review

**DOI:** 10.3389/fendo.2021.666791

**Published:** 2021-04-30

**Authors:** Xiaohai Liu, Renzhi Wang, Mingchu Li, Ge Chen

**Affiliations:** ^1^ Department of Neurosurgery, Xuanwu Hospital Capital Medical University, Beijing, China; ^2^ Chinese Pituitary Specialists Congress, Beijing, China; ^3^ Department of Neurosurgery, Peking Union Medical College Hospital, Chinese Academy of Medical Sciences and Peking Union Medical College, Beijing, China

**Keywords:** IgG4-related disease, IgG4-related inflammatory pseudotumor, clivus, endoscopic transsphenoidal approach, case report

## Abstract

IgG4-related inflammatory pseudotumors are very uncommon and are characterized histologically by the presence of inflammatory swellings with increasing IgG4-positive plasma cells and lymphocytes infiltrating the tissues. As reports of intracranial IgG4-related pseudotumors are very rare, we report a case of an IgG4-related inflammatory pseudotumor involving the clivus mimicking meningioma. A 46-year-old male presented with intermittent headache for 2 years and a sudden onset of dysphagia and dysphonia of 7 days’ duration along with lower limb weakness. Enhanced magnetic resonance imaging (MRI) of the skull base revealed an isointense signal on T1- and T2-weighted images from an enhanced mass located at the middle of the upper clivus region, for which a meningioma was highly suspected. Then, an endoscopic transsphenoidal approach was adopted and the lesion was partially resected, as the subdural extra-axial lesion was found to be very tough and firm, exhibiting fibrous scarring attaching to the brain stem and basal artery. After the surgery, brain stem and posterior cranial nerve decompression was achieved, and the patient’s symptoms, such as dysphagia, dysphonia and lower limb weakness, improved. Pathological findings showed many IgG4-positive plasma cells and lymphocytes surrounded by collagen-rich fibers. The patient was sent to the rheumatology department for further glucocorticoids after the diagnosis of an IgG4-related inflammatory pseudotumor was made. This case highlights the importance of considering IgG4-related inflammatory pseudotumors as a differential diagnosis in patients with lesions involving the clivus presenting with a sudden onset of symptoms of dysphagia and dysphonia along with lower limb weakness when other more threatening causes have been excluded. IgG4-related inflammatory pseudotumors are etiologically enigmatic and unpredictable, and total resection might not be warranted. Glucocorticoids are usually the first line of treatment after diagnosis.

## Introduction

Although intracranial hypophysitis is well known and relatively common, IgG4-related disease (IgG4-RD) is very rare and characterized histologically by the presence of acute or chronic inflammatory swellings of involved organs with increasing IgG4-positive plasma cells and lymphocytes infiltrating the tissues (1). Although several cases of IgG4-related inflammatory pseudotumors have been reported, there are few reports of intracranial IgG4-related inflammatory pseudotumors (2–14). The preoperative diagnosis of the disease is very important as IgG4-related inflammatory pseudotumors are more likely sensitive to steroid therapy but not surgery. Here, we report a rare case of an IgG4-related inflammatory pseudotumor in the middle of the upper clivus region, mimicking meningioma, and review the literature on such cases (2–14). We hope to highlight the importance of considering IgG4-related inflammatory pseudotumors as a differential diagnosis in patients presenting with clivus lesions.

## Case Presentation

### Medical History

**Figure 1:**
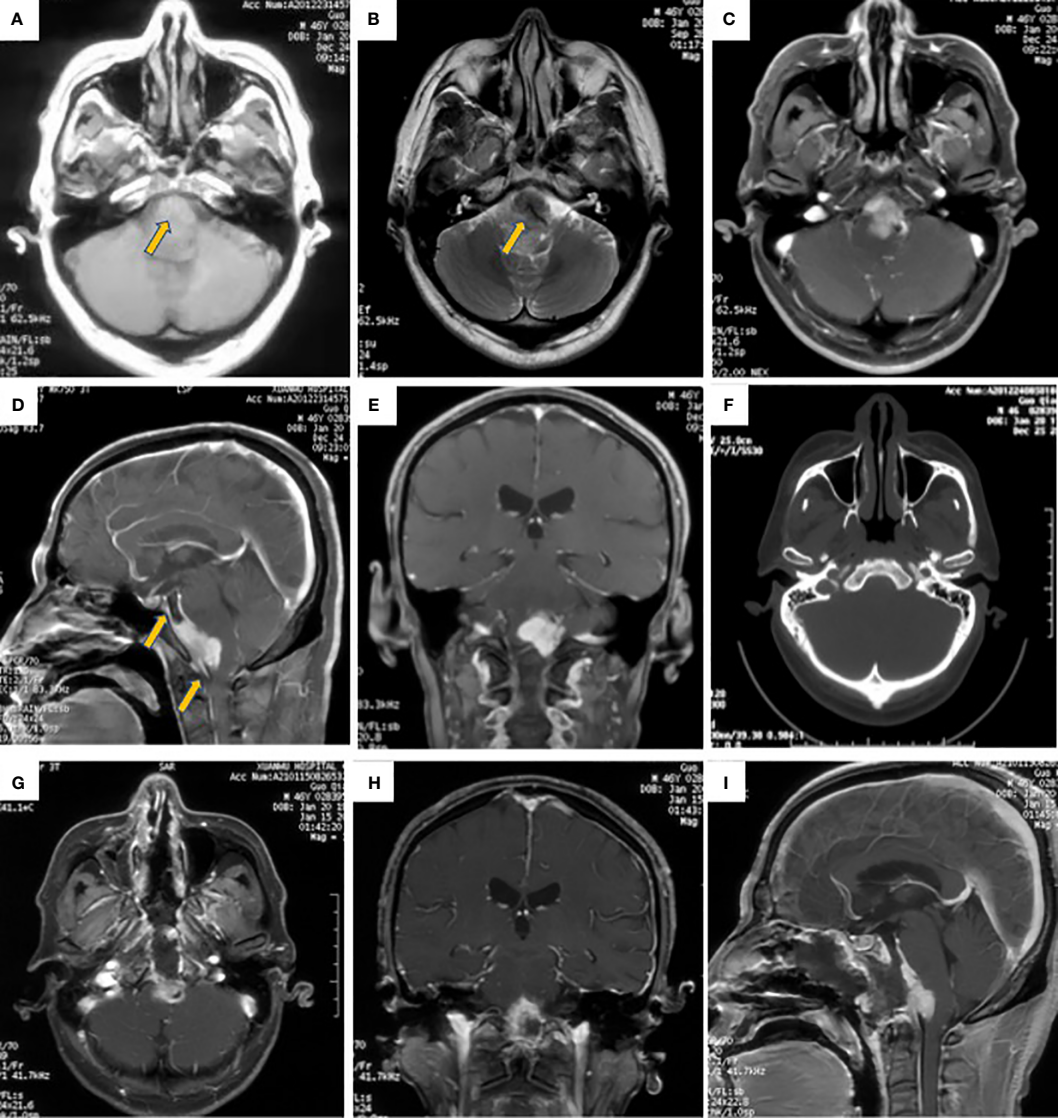
Brain MRI images showed intracranial mass located at middle of upper clivus region. **(A, B)**. The lesion located at middle of upper clivus region presented with isointense signal in T1-weighted MRI image (**A**, the arrow), and hypointense signal in T2-weighted MRI image (**B**, the arrow); **(C, E)**: The mass was uniformly enhanced after contrast MRI scan, and the base of the tumor was extensively stick to the adjacent dura and brain stem, showing meningeal tail sign (the arrow in **D**), and meningioma was highly suspected. **(F)**. CT bone window showed no bone invasion. **(G, I)**. The MRI showed partial resection of the lesion.

The patient was a 46-year-old male who presented with intermittent headache for 2 years and a sudden onset of dysphagia and dysphonia of 7 days’ duration along with lower limb weakness upon physical examination. His visual acuity and fields for both eyes were relatively normal. He had no past history of autoimmune disease or other illness. Magnetic resonance imaging (MRI) of the skull base revealed a predominantly hypointense signal on T1- and T2-weighted images from a mass located in the middle of the upper clivus area, and it was homogenously enhanced after contrast MRI; meningioma was highly suspected (**Figures 1A–F**). Routine laboratory tests showed no abnormal data. The levels of pituitary hormones were all within normal limits. The study was approved by the Research Ethics Committee of our hospital and written informed consent was obtained from the patient according to institutional guidelines for publication of this case report and any accompanying images.

### Surgical Details

**Figure 2:**
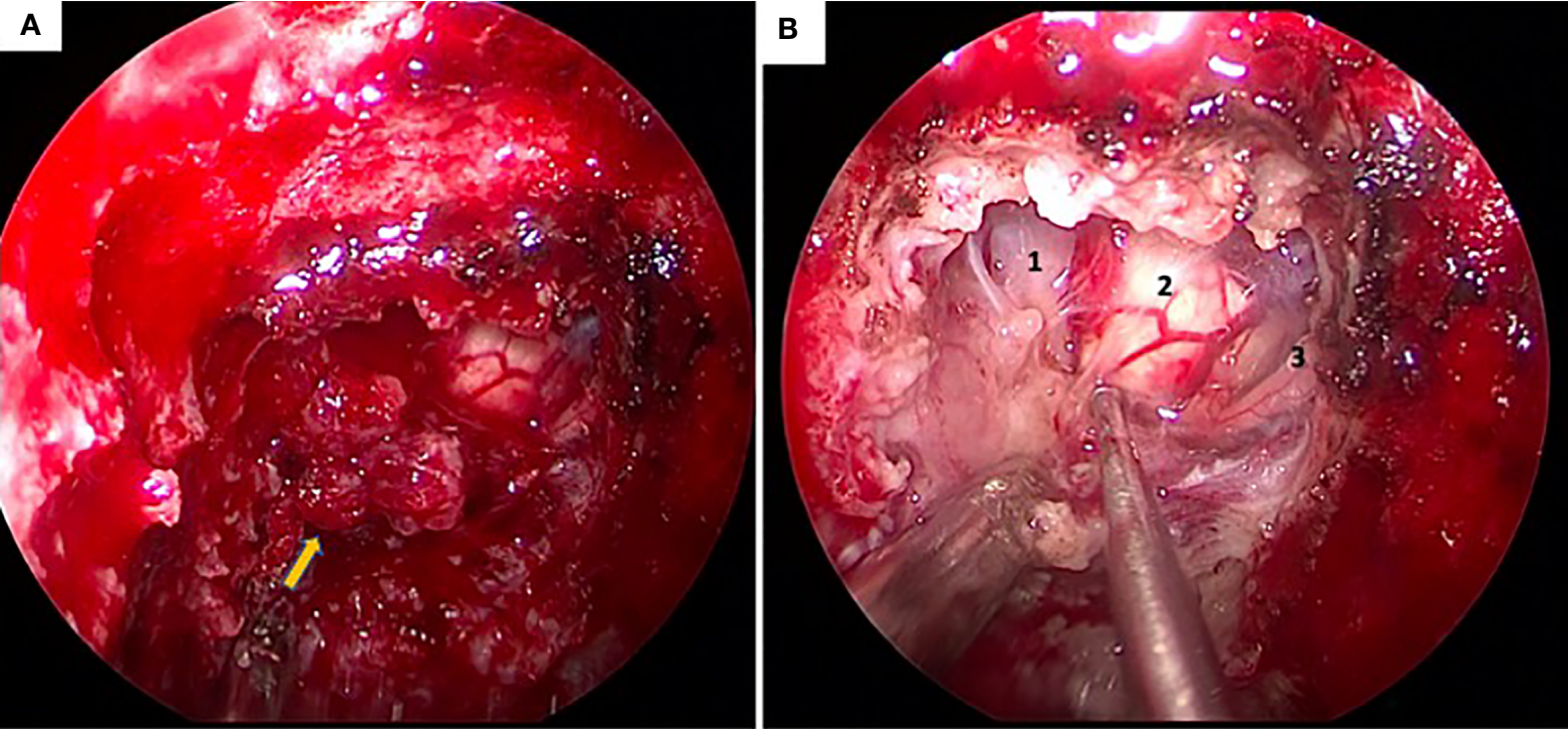
Intra-operative condition of the lesion located at middle of upper clivus region. **(A)** the lesion was very tough and firm (the arrow); **(B)** the lesion was stick to the brain stem and basal artery (1: brain stem; 2: basal artery; 3: abducent nerve).

On Dec. 30, 2020, the patient underwent endoscopic transsphenoidal surgery. During the operation, the extra-axial lesion was found to be subdural and was very tough and firm, exhibiting fibrous scarring attaching the mass to the brain stem (**Figures 2A, B**). Because the lesion did not resemble meningioma in texture, we sent some tumor tissue for intraoperative frozen tissue pathology, which showed many lymphoplasmacytes surrounded by collagen-rich fibers. As inflammatory lesions may be sensitive to glucocorticoids, the lesion was partially resected owing to its tenacious texture and close adhesion to the brain stem (**Figures 1G–I**). Postoperatively, brain stem and posterior cranial nerve decompression was achieved. Symptoms, such as dysphagia and dysphonia along with lower limb weakness, were improved, and the patient did not suffer from additional neurological deficits.

### Postoperative Treatment

**Figure 3:**
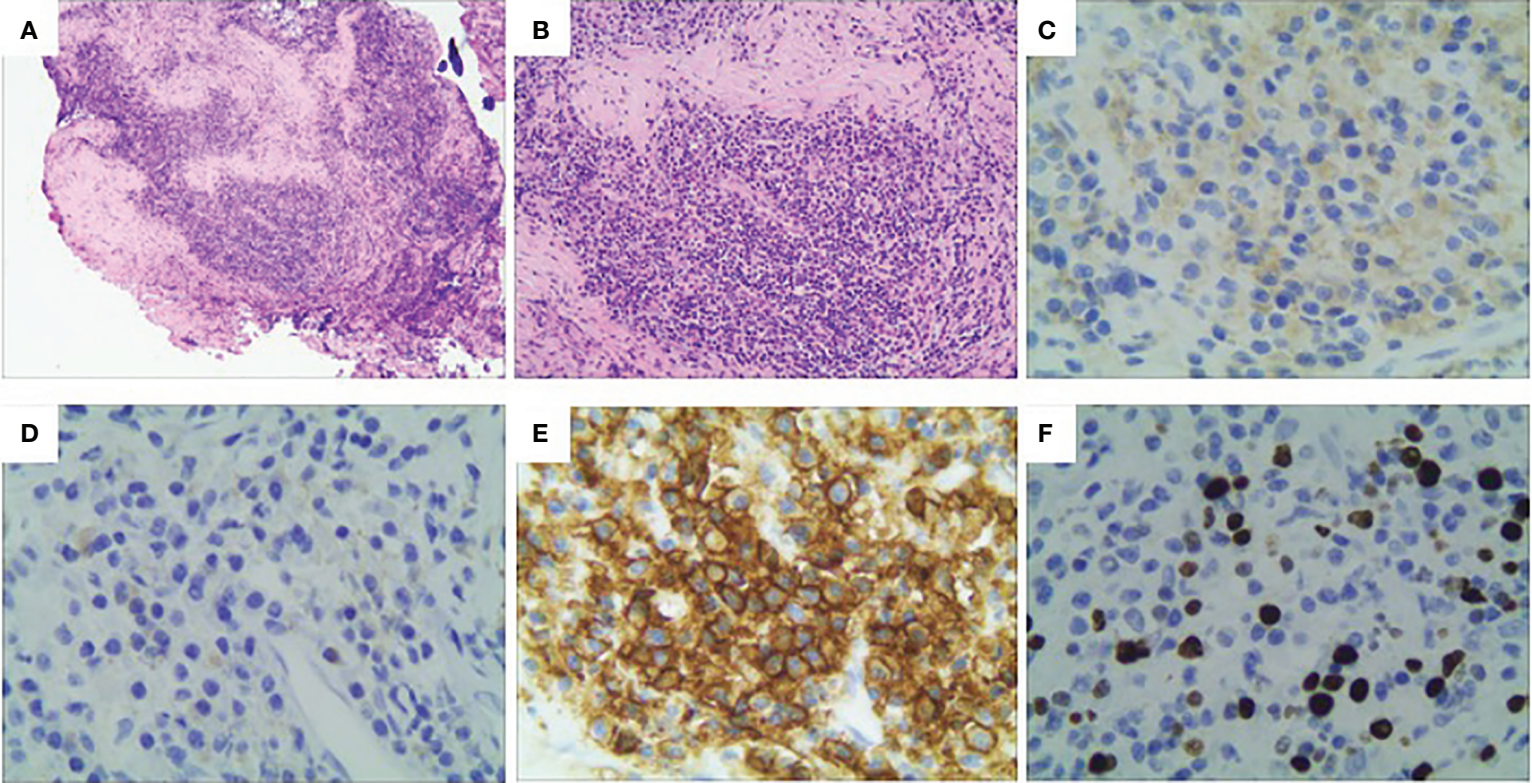
Histological features of the lesion revealed an IgG4-related inflammatory pseudotumor. **(A, B)** Hematoxylin and eosin (H&E) stain showed the lesion was composed of sclerosing fibrosis associated with dense lymphoplasmacytic infiltration (A: ×40; B:×100); **(C, D)** Immunohistochemical analysis revealed over 400 IgG4-positive cells per high-powered field and a high IgG4/IgG ratio (C, IgG, ×200; D, IgG4, ×200). **(E)** Immunohistochemical staining of CD38; **(E)** Immunohistochemical staining of Ki-67. **(F)** Immunohistochemical staining of Ki-67.

The histopathological findings revealed an inflammatory pseudotumor involving the clivus, with sclerosing fibrosis mixed with lymphocytes and IgG4-positive plasma cells infiltrating into the tissues (**Figure 3**). Hematoxylin and eosin (H&E) stain showed the lesion was composed of sclerosing fibrosis associated with dense lymphoplasmacytic infiltration (**Figures 3A, B**). Immunohistochemical analysis revealed over IgG4-positive cells per high-powered field and a high IgG4/IgG ratio (**Figures 3C, D**). Immunohistochemistry revealed positivity for vimentin, EMA, CD3, CD20, CD38, CD68, kappa, lambda and IgG4 but negativity for PAS and CD34. and the Ki-67 index was 10% (**Figures 3E, F**). The blood sample for further immune disease investigations showed no elevated levels of IgG (12.5 g/L; reference range 7.51-15.6 g/L), IgA (2.34 g/L; reference range 0.82-4.53 g/L), IgM (0.68 g/L; reference range 0.46-3.04 g/L) or IgE (131.0 IU/ml; reference range 5-165 g/L) in the serum. Subsequent ultrasonography and computed tomography (CT) for thorax and abdomen were performed, and no other lesions were detected. After a discussion of MDT (Multidisciplinary Team), the diagnosis of IgG4-related inflammatory pseudotumor was made, and the patient was sent to the rheumatology department for further glucocorticoid treatment.

## Discussion

IgG4-RD is a chronic inflammatory lesion involving many organs of unknown origin (15). An epidemiological study revealed an estimated prevalence in the Japanese population of 6 cases per 100,000 people (16). There are several alternative terms for IgG4-related inflammatory lesions, such as inflammatory pseudotumors, inflammatory myofibroblastic tumors, plasma cell granulomas or pseudotumors, xanthomatous disease, pseudosarcomatous myofibroblastic diffusion, and inflammatory myofibrohistiocytic myofibroblastoma (17). According to the consensus statement on the pathology of IgG4-related disease, the diagnosis of IgG4-RD is based on three histological criteria: dense lymphoplasmocytic infiltrate, fibrosis arranged at least focally in a storiform pattern and obliterative phlebitis (18). Interestingly, serum IgG4 levels are normal in approximately 40% of patients with biopsy-proven IgG4-RD. Therefore, increased serum IgG4 levels are not necessary for the diagnosis of IgG4-related inflammatory pseudotumors. In our case, the diagnosis of IgG4-related inflammatory pseudotumors was based on sclerosing fibrosis mixed with lymphocytes and IgG4-positive plasma cells infiltrating into the tissues, while the serum IgG4 level was normal in our case.

The most common organ affected by IgG4-related inflammatory pseudotumors is the pancreas, but it can also involve the biliary tract, salivary and lacrimal glands, kidneys, orbital tissues, lymph nodes, lungs and many others (18). IgG4-related inflammatory pseudotumors have been found to involve, in rare cases, the nervous system. For lesions in the nervous system, IgG4-RD is relatively common in the orbit and pituitary and is characterized by a variety of histological manifestations (19). Based on previous reports (2–14), MR of the lesion usually demonstrated iso-signal intensity in T1-weighted imaging and low or iso-signal intensity in T2-weighted imaging, and intense enhancement was observed after gadolinium-enhanced T1-weighted imaging. Therefore, it is difficult to determine whether an infiltrating soft tissue mass is a IgG4-related inflammatory pseudotumor, a malignant tumor or a meningioma. For example, in our case, the tumor was initially thought to be a meningioma. In the absence of symptoms or signs of infection such as fever, the clinical manifestations varied based on the locations.

For our patients, the lesion was located in the middle of the upper clivus area. Based on the symptoms and MRI manifestation, a meningioma was highly suspected. Then, the endoscopic transsphenoidal approach was adopted, and the mass was partially resected, as the subdural extra-axial lesion was found to be very tough and firm, exhibiting fibrous scarring attaching the mass to the brain stem. Intraoperative frozen pathological findings showed many lymphoplasmacytes surrounded by collagen-rich fibers. The histopathological findings revealed an inflammatory pseudotumor involving the clivus, with sclerosing fibrosis mixed with lymphocytes and plasma cells in which the immunohistochemistry was positive for IgG4.

Although twenty-six cases of intracranial pseudotumors due to IgG4-RD have been described (**Table 1**) (2–14), the pathogenesis of IgG4-associated inflammatory pseudotumors is still unclear; nevertheless, multiorgan system conditions with pathological features that are largely consistent across a wide range of organ systems are recognized. The convex surface of the brain, the parasagittal falx, and the base of the skull (such as the sphenoid crest region) are common sites for meningiomas, but, as shown in this case, IgG4-related inflammatory pseudotumors are also possible, highlighting the importance of considering IgG4-related inflammatory pseudotumors as a differential diagnosis in these patients with lesions involving these structures.

**Table 1:** Reported cases of IgG4-related intracranial pseudotumors.

	Author	Sex	Age	Lesion	Intracranial multiple lesions	Extracranial lesions	Tumor consistency	Encasing artery
1	Lui P.C (6).	F	52	rt. Lateral ventricle	–	–	n.d.	n.d.
2	Lui P.C (6).	M	45	dura at rt. frontal region	–	–	n.d.	n.d.
3	Lui P.C (6).	F	26	dura at lt. frontotemporal region	–	–	n.d.	n.d.
4	Okano (20)	M	62	rt. Meckle’s cave and lt. foramen magnum	+	+	**hard**	n.d.
5	Lindstrom (7)	M	53	posterior fossa	–	–	n.d.	n.d.
6	Lindstrom (7)	F	54	dura at petrous apex	–	–	n.d.	n.d.
7	Lindstrom (7)	F	51	sella turcica	–	–	n.d.	n.d.
8	Kim (3)	M	43	lt. frontal area and near corpus callosum	+	–	**hard**	n.d.
9	Katsura (5)	F	59	along trigeminal nerve	–	–	n.d.	n.d.
10	Wong (21)	M	77	tumor at pituritory	–	+	**hard**	n.d.
11	Nishino (2)	M	67	dura-based mass in the Sylvian fissures, bilateral trigeminal nerves and pituitary stalk	+	+	non-surgery case	**attaching to bilateral ICA in MRI**
12	Noshiro (4)	M	39	optic nerve	–	+	n.d.	n.d.
13	Katsura (10)	M	64	orbital apex and cavernous sinus	–	+	n.d.	n.d.
14	Katsura (10)	F	61	cavernous sinus to pterygopalatine fossa, infraorbital canal	–	–	n.d.	n.d.
15	Katsura (10)	M	55	cavernous sinus to orbit	–	–	n.d.	n.d.
16	Katsura (10)	M	65	cavernous sinus to pterygopalatine fossa	–	+	n.d.	n.d.
17	Katsura (10)	M	62	anterior clinoid process and posterior fossa dura mater	–	+	n.d.	**VA penetrating the mass**
18	Moss (9)	F	36	middle cranial fossa, foramen magnum and superior frontal	+	–	n.d.	**involving cavernous sinus**
19	Moss (9)	F	50	middle and anterior cranial fossa	–	–	n.d.	**involving cavernous sinus**
20	Rice (11)	M	46	carotid canal, jugular foramen and foramen ovale	–	+	n.d.	n.d.
21	Goulam-Houssein (12)	M	70	anterior temporal lesion	–	–	n.d.	n.d.
22	Goulam-Houssein (12)	M	54	cavernous sinus and Meckel’s cave	+	–	n.d.	**smooth narrowing cavernous ICA**
23	Goulam-Houssein (12)	F	28	suprasellar mass	–	–	n.d.	**occlusion of ICA**
24	Kuroda (13)	F	72	around medulla, cerebellum and middle fossa	+	–	hard	encasing VA
26	Tang (14)	F	50	right upper clivus area	–	–	soft	n.d.
**27**	**Our case**	F	50	**Middle upper clivus area**	–	–	**hard**	**attaching to BA**

Bold values represent our case.

n.d., not described.

In terms of treatment, glucocorticoids are the standard treatment once the diagnosis of IgG4-related inflammatory pseudotumors is made (18). However, the diagnosis is based on the pathology from a biopsy or surgery. After diagnosis, glucocorticoids have been used in several cases of IgG4 intracranial pseudotumors, and nearly all the patients achieved remission or tumor reduction (2, 3, 9, 12, 13). Although recurrence despite after corticosteroid prescription has been reported in the literature, prognosis of the patients with IgG4-related related inflammatory pseudotumor was good. If patients are not steroid responsive, local radiation therapy has been shown to be effective in some cases (18). Therefore, our patient with residual pseudotumor was sent to the rheumatology department for further glucocorticoid treatment as soon as the diagnosis of IgG4-related inflammatory pseudotumor was made.

## Conclusion

This case showed IgG4-related inflammatory pseudotumors involving the clivus, mimicking meningioma, highlighting the importance of considering IgG4-related inflammatory pseudotumors as a differential diagnosis in patients presenting with a sudden onset symptoms of dysphagia and dysphonia along with lower limb weakness when other more threatening causes were excluded. Inflammatory pseudotumors are etiologically enigmatic and unpredictable, and total resection might not be warranted. Glucocorticoids are usually the first line of treatment after diagnosis.

## Data Availability Statement

The original contributions presented in the study are included in the article/supplementary material. Further inquiries can be directed to the corresponding author.

## Ethics Statement 

The studies involving human participants were reviewed and approved by Research Ethics Committee of Xuanwu Hospital, Capital Medical university. The patients/participants provided their written informed consent to participate in this study. Written informed consent was obtained from the individual(s) for the publication of any potentially identifiable images or data included in this article.

## Author Contributions

All these four authors were involved in patient treatment, data collection and analysis, and manuscript writing. All authors contributed to the article and approved the submitted version.

## Funding

The financial support for this study was provided by the Scientific Research Project of Capital Health Development in 2018 (grant number: 2018-4-4018). The funding institutions played no role in the design of the study, data collection or analysis, decision to publish, or preparation of the manuscript.

## Conflict of Interest

The authors declare that the research was conducted in the absence of any commercial or financial relationships that could be construed as a potential conflict of interest.
